# Protocol for the PINCER trial: a cluster randomised trial comparing the effectiveness of a pharmacist-led IT-based intervention with simple feedback in reducing rates of clinically important errors in medicines management in general practices

**DOI:** 10.1186/1745-6215-10-28

**Published:** 2009-05-01

**Authors:** Anthony J Avery, Sarah Rodgers, Judith A Cantrill, Sarah Armstrong, Rachel Elliott, Rachel Howard, Denise Kendrick, Caroline J Morris, Scott A Murray, Robin J Prescott, Kathrin Cresswell, Aziz Sheikh

**Affiliations:** 1 Division of Primary Care, The Medical School, Queen's Medical Centre, Nottingham, NG7 2UH, UK; 2 Division for Social Research in Medicines and Health, The School of Pharmacy, University of Nottingham, University Park, Nottingham, NG7 2RD, UK; 3 Drug Usage & Pharmacy Practice Group, School of Pharmacy & Pharmaceutical Sciences, University of Manchester, Oxford Road, Manchester, M13 9PL, UK; 4 Trent Research Design Service, Division of Primary Care, Tower Building, University Park, Nottingham, NG7 2RD, UK; 5 School of Pharmacy, University of Reading, PO Box 226, Whiteknights, Reading, RG6 6AP, UK; 6 Department of Primary Health Care and General Practice, Wellington School of Medicine and Health Sciences, University of Otago, Mein Street, Wellington South, New Zealand; 7 Centre for Population Health Sciences, University of Edinburgh, 20 West Richmond Street, Edinburgh, EH8 9DX, UK

## Abstract

**Background:**

Medication errors are an important cause of morbidity and mortality in primary care.

The aims of this study are to determine the effectiveness, cost effectiveness and acceptability of a pharmacist-led information-technology-based complex intervention compared with simple feedback in reducing proportions of patients at risk from potentially hazardous prescribing and medicines management in general (family) practice.

**Methods:**

***Research subject group***: "At-risk" patients registered with computerised general practices in two geographical regions in England.

***Design***: Parallel group pragmatic cluster randomised trial.

**Interventions**: Practices will be randomised to either: (i) Computer-generated feedback; or (ii) Pharmacist-led intervention comprising of computer-generated feedback, educational outreach and dedicated support.

***Primary outcome measures***: The proportion of patients in each practice at six and 12 months post intervention:

- with a computer-recorded history of peptic ulcer being prescribed non-selective non-steroidal anti-inflammatory drugs

- with a computer-recorded diagnosis of asthma being prescribed beta-blockers

- aged 75 years and older receiving long-term prescriptions for angiotensin converting enzyme inhibitors or loop diuretics without a recorded assessment of renal function and electrolytes in the preceding 15 months.

***Secondary outcome measures***; These relate to a number of other examples of potentially hazardous prescribing and medicines management.

***Economic analysis***: An economic evaluation will be done of the cost per error avoided, from the perspective of the UK National Health Service (NHS), comparing the pharmacist-led intervention with simple feedback.

***Qualitative analysis***: A qualitative study will be conducted to explore the views and experiences of health care professionals and NHS managers concerning the interventions, and investigate possible reasons why the interventions prove effective, or conversely prove ineffective.

***Sample size***: 34 practices in each of the two treatment arms would provide at least 80% power (two-tailed alpha of 0.05) to demonstrate a 50% reduction in error rates for each of the three primary outcome measures in the pharmacist-led intervention arm compared with a 11% reduction in the simple feedback arm.

**Discussion:**

At the time of submission of this article, 72 general practices have been recruited (36 in each arm of the trial) and the interventions have been delivered. Analysis has not yet been undertaken.

**Trial registration:**

Current controlled trials ISRCTN21785299

## Background

Medication errors are an important cause of morbidity and mortality in primary and secondary care and a number of reports from the UK, USA and other countries have highlighted the need to reduce error rates to prevent patients suffering from avoidable harm[1,2].

In England, publication by the Government of *An organisation with a memory*[1] and *Building a safer NHS for patients *[3] illustrates a strong commitment to reducing errors; the establishment of the National Patient Safety Agency (NPSA) was a clear example of this commitment.

Recent UK government reports have suggested that while there may still be a need to understand more about medication errors and the reasons for their occurrence[3,4], the priority now must be to find effective, acceptable and sustainable ways of preventing patients from being harmed as a result of errors.

### Definition of error

In this study we have taken the definition of "medication error" used by the US National Co-ordinating Council for Medication Error Reporting and Prevention[5] and the NPSA:

*"A medication error is any preventable event that may cause or lead to inappropriate medication use or patient harm while the medication is in the control of health professional, patient or consumer"*.

This definition thus covers the whole of the medicines management process, from prescribing through to medication monitoring[4].

### Human error theory

To understand the causes of errors it is helpful to have an underlying theoretical framework. James Reason's work in this field has had a major influence on our understanding of the causes of medication errors[6]. We have, in developing our interventions, taken account of human error theory in considering the causes of medication errors in primary care and the approaches that are most likely to reduce error rates.

### Medication errors in primary care

This proposal builds on our narrative[7] and systematic[8,9] reviews of the international literature on medication errors in primary care and on our own related empirical work [10-12]. We have drawn on these experiences to identify and select outcome measures that are clinically important. This work has shown that the following groups of drugs are both commonly and consistently associated with medication errors that result in serious morbidity and, in some cases, mortality:

• Cardiovascular drugs (including angiotensin converting enzyme (ACE) inhibitors, beta-adrenoceptor blocking drugs and diuretics)

• Non-steroidal anti-inflammatory drugs (NSAIDs)

• Oral anticoagulants (warfarin).

We have also taken account of errors associated with the use of methotrexate, in view of warnings about this drug from the Chief Pharmaceutical Officer for England[4], and lithium and amiodarone because of their narrow therapeutic indices and the need to undertake regular blood test monitoring[13].

### Underlying causes of medication errors

There have been a number of studies that have investigated the underlying causes of medication errors in hospitals[14,15]. Leape *et al*, for example, identified 16 major systems failures from an analysis of 334 errors[14]. The most common underlying problem was "failure of drug knowledge dissemination" (i.e. the doctor not knowing enough about the drug) and this accounted for 29% of errors[14]. In contrast, Dean *et al *investigated the causes of 44 prescribing errors and found that slips in attention or failure to apply relevant rules were the commonest underlying causes[15].

There have been relatively few detailed analyses of the causes of medication errors in primary care although several studies have identified the points in the medicines management process where most errors occur[10,16]

Gurwitz *et al *found that the majority of preventable adverse drug events associated with community-based prescribing were due to errors in the prescribing and monitoring phases of pharmaceutical care[16]. These findings were mirrored in the study of drug-related hospital admissions that was undertaken in Nottingham, UK[10], whereby 35% of admissions were thought to be due to unsafe prescribing decisions and 26% due to inadequate monitoring.

Our analysis of these studies suggest that in aiming to reduce rates of medication error in primary care the key factors that need to be addressed are:

• ensuring that general (family) practitioners (GPs) are aware of the risks of the drugs most commonly associated with adverse events

• ensuring that GPs recognise the hazards of rule violation, e.g. prescribing drugs that are contraindicated

• developing robust systems for monitoring patients on high-risk medications (including call and recall for blood tests) so that patients are not exposed to correctable hazards.

We have taken account of these issues in the design of the complex pharmacist-led information-technology (IT) based intervention for our trial.

### The development of methods for identifying medication errors using GP computer systems

The use of clinical computer systems to identify patients with medication errors is a potentially powerful method for "error trapping" that may allow general practices to correct errors before patients are harmed.

We used MIQUEST[17] software successfully in our pilot work to identify preventable drug-related morbidity in general practice[18,19]. This process involved writing precise computer queries that are capable of extracting the information required.

In our pilot work[18,19], we found the processing of MIQUEST data very time-consuming. This is because it usually involves visiting general practices to extract data and then a considerable amount of work in processing and checking the data. Also, it does not produce user-friendly output for practices on individual patients who are deemed to be "at risk".

We resolved these problems through the use of an additional type of software called Quest Browser . This well-established software uses MIQUEST queries of GP computer systems, but has several advantages over using MIQUEST alone. Firstly, it can produce user-friendly feedback at the practice-level on patients with medication errors (or any other clinical problem). Secondly, output from Quest Browser can be imported straight into a database without the need for additional manipulation. Thirdly, Quest Browser has a facility (called Quest Browser Central) whereby, with agreement from the practices and research ethics committees, linked-anonymised data can be sent to researchers in an encrypted form via the Internet. This reduces the number of visits that researchers need to make to practices and helps with the timely collection of data.

### Development of the complex pharmacist-led IT-based intervention

Informed by the Medical Research Council's (MRC) framework for complex interventions[20], we took account of the theoretical considerations outlined above, along with pilot work, to develop the pharmacist-led IT-based intervention. This is described in more detail below (see "pharmacist-led intervention" section).

### Aims of the study

To determine the effectiveness, cost effectiveness and acceptability of a complex pharmacist-led IT-based intervention compared with simple feedback in reducing proportions of patients at risk from potentially hazardous prescribing and medicines management in general practice.

### Specific objectives

1. To test the hypothesis that a pharmacist-led IT-based complex intervention using educational outreach and practical support is more effective than simple feedback in reducing the proportion of patients at risk from hazardous prescribing and medicines management in general practice.

2. To conduct an economic evaluation of the cost per error avoided, from the perspective of the NHS, of the pharmacist-led intervention compared with simple feedback.

3. To explore the views and experiences of health care professionals and NHS managers concerning the intervention; investigate the possible reasons why the interventions might prove effective or ineffective, and inform future roll-out of the pharmacist-led intervention if it proves to be effective.

## Methods/Design

### Trial design

We will conduct a two-arm pragmatic cluster randomised trial. Trial practices will receive either i) computerised feedback on patients identified to be at risk from potentially hazardous prescribing and medicines management or ii) a complex pharmacist-led IT-based intervention in addition to computerised feedback.

### Eligibility of general practices for entering the trial

#### Inclusion criteria

• NHS general practices within a 50 mile radius of Manchester and Nottingham in England.

• Practices within NHS primary care trusts (PCTs) that agreed to be involved in the study.

• Practices that were laboratory-linked (or had other reliable systems for recording blood test results on the practice computer system) for at least 15 months prior to the time of baseline data collection (being laboratory-linked means having all blood test results relayed electronically to the practice so that these can be downloaded into patients' computerised records).

• Practices that agreed to participate in the study.

#### Exclusion criteria

• Practices that stated they did not routinely record morbidities such as asthma or peptic ulcer on patients' computerised records.

• Practices not routinely using their computers to record prescriptions issued.

• Practices that were intending to change their GP computer systems to that of a different supplier which was not Quest Browser compatible during the course of the study.

• Practices in PCTs that were undertaking interventions that might overlap with our intervention.

• Practices that were involved in the pilot study for the trial.

• Practices that expected major changes in list size (numbers of registered patients) during the course of the study, either because of the splitting up of the practice, merger with other practices or any other reason for a large influx or loss of patients.

### Recruitment

#### General practices

We wrote to general practices in PCTs in Nottinghamshire, Staffordshire and Central and Eastern Cheshire, England informing them of the study. Where practices expressed an interest in participating we arranged a face-to-face meeting at which the study was explained in more detail. A member of the practice team then signed a consent form if the practice decided to participate. Seventy-two general practices were recruited.

#### Patients

For the purposes of the health economic analysis, the general practices recruited to the study were asked to write to all patients identified through baseline data collection who appeared in the numerator of one of our outcome measures (i.e. they had potentially been subjected to hazardous prescribing or medicines management). In the accompanying patient information leaflet, patients were given information about the study and were asked to give consent for the research team to access their medical records. Patients were asked to sign a consent form and to return this to the study team.

For outcome measure six (see below for further details), we identified some practices that keep their records of international normalised ratio (INR) results for monitoring anticoagulation therapy separate from their main practice computer system and thus appeared to have very high proportions of patients not having INRs checked according to the computer searches we undertook. In these cases, we did not write to patients to seek consent to access their records because it is likely that the majority were not at risk.

#### Participants for the qualitative analysis

In order to obtain a range of perspectives, purposive sampling has been employed. This has enabled us to identify a range of NHS staff (e.g. clinicians and administrative staff) working in both the intervention and control practices in both geographical regions and in practices of varying size. Also, on the basis of suggestions from trial researchers and intervention pharmacists, we have recruited participants with a range of viewpoints, including those from practices where the interventions appear to have been received positively and those where problems have been encountered.

Other key stakeholders we have recruited include prescribing and clinical governance leads of primary care organisations, trial pharmacists, community pharmacists and researchers closely involved in the roll-out of the study.

We wrote to potential participants for the qualitative analysis and obtained written consent before undertaking interviews.

### Interventions

#### Simple feedback

Those practices randomly allocated to this arm received computerised feedback on patients identified to be at risk from potentially hazardous prescribing and medicines management along with brief written educational materials explaining the importance of each type of error. This information was given to a nominated member of the general practice (usually the practice manager) after baseline data have been collected from the practice computer system, using Quest Browser software.

Practices in the simple feedback arm were asked to try to make any changes to patients' medications within a 12 week (intervention) period following the baseline data collection.

#### Pharmacist intervention

Those practices randomly allocated to this arm received simple feedback and in addition, had a complex pharmacist-led IT-based complex intervention.

First, the pharmacist arranged to meet with members of the practice team to discuss the computer-generated feedback on patients with medication errors. All doctors were encouraged to attend this meeting along with at least one member of the nursing staff, the practice manager and at least one member of the reception staff.

Before the meeting, wherever possible, all relevant members of staff were provided with a brief summary of the objectives of the pharmacist-led intervention and a summary of the findings from the computer search.

At the meeting the pharmacists were asked to use the following approach derived from the principles of educational outreach[21] while also taking account of human error theory[6]:

• Establish professional credibility by explaining their own background in clinical pharmacy and their affiliation with either the University of Manchester or University of Nottingham (depending on the site they are working from)

• Take a non-judgemental approach in all discussions with members of the practice team

• Outline the findings from the computer search

• Explore the views of team members about the findings

• Investigate the baseline knowledge of team members regarding the importance of each of the errors

• Provide clear, concise, evidence-based materials on each of the errors, encouraging active participation by team members

• Explore the views of team members on the underlying causes of the medication errors (using root-cause analysis techniques[22] where appropriate)

• Explain their availability to work part-time with the practice over the following 12 weeks to:

- Help take corrective action in individual patients with medication errors

- Help improve the systems operating in the practice in order to prevent future errors.

• Encourage the team to agree on an action plan with clear objectives

• Ask for a member of the practice team to volunteer to liaise with the pharmacist over arrangements for making changes to individual patients' medication and introducing changes to systems within the practice

• Ask the practice to agree to a follow up meeting within four to six weeks of the initial meeting.

Following this initial meeting, the pharmacists were expected to use a range of techniques to help correct the medication errors that had been identified and prevent future medication errors. They were expected to work closely with the practice team member assigned to provide liaison with other members of the practice.

We envisaged that the pharmacists would be taking any, or all, of the following approaches to deal with patients identified to be at risk from potentially hazardous prescribing and medicines management:

• Inviting patients into the surgery for a prescription review with the pharmacist, or a member of the general practice team, with the aim of correcting medication errors, e.g.

- For patients with a past history of peptic ulcer who were being prescribed a non-selective NSAID to either:

▪ Stop the NSAID

▪ Add a proton pump inhibitor (PPI)

▪ Consider using a COX-2 inhibitor, while recognising concerns about these drugs in relation to cardiovascular risk.

- For patients with asthma who were being prescribed a beta-blocker:

▪ In those taking beta-blocker eye drops for glaucoma, to change to an alternative preparation

▪ In those taking oral beta-blockers, to carefully consider the risks and benefits of the medication and, where appropriate, slowly withdraw the drug and replace it with an alternative preparation.

- For patients who were being prescribed methotrexate without instructions that it should be taken weekly:

▪ Carefully check the dosage instructions

▪ Convey this information to the patient verbally and in writing

▪ Ensure that accurate dosage instructions were entered onto the computer system so that these would be printed out when the drug was next issued.

• Inviting the following groups of patients to have a blood test:

- Those aged 75 years and older being prescribed ACE inhibitors or loop diuretics who had not had a blood test to check renal function and electrolytes within the previous 15 months

- Those being prescribed methotrexate who had not had a full blood count or liver function test within the previous three months

- Those being prescribed warfarin who had not had an INR test within the previous 12 weeks (this is the maximum interval recommended by the British National Formulary[23]).

We envisaged the pharmacists taking the following approaches to try to *prevent *future instances of hazardous prescribing and medicines management, having agreed these approaches with the practice teams:

• In relation to hazardous prescribing:

- Meeting up with any doctors unable to attend the initial meeting in order to provide educational outreach

- Reinforcement of educational messages provided at the initial meeting by repeating these messages at future meetings

- Encouraging doctors to take heed of contraindication messages on their computer systems.

• In relation to inadequate blood-test monitoring:

- Encouraging practices to use their computer systems to automatically recall patients for a blood test if they have gone beyond a pre-specified time

- To use routine prescription reviews as the trigger for ensuring that if patients need blood tests, these are arranged.

Throughout the intervention period the pharmacists were asked to maintain regular contact with the practice liaison member of staff to facilitate changes and discuss, and resolve, any difficulties encountered. The pharmacists were asked to keep a written log of changes made in relation to patients with medication errors, and changes made to practice systems.

Towards the end of the intervention period, the pharmacists were asked to undertake a further check of patients' computer records to provide feedback to practices on progress made in correcting medication errors. They were asked to arrange a final meeting with members of the practice team to:

• Provide feedback on progress made in dealing with patients identified to be at risk from potentially hazardous prescribing and medicines management

• Provide feedback on changes made to safety systems

• Reinforce key educational messages

• Agree on an action plan for the practice to continue to work towards reducing instances of hazardous prescribing and medicines management.

### Allocation of trial interventions

The practice was the unit of allocation. Eligible GP practices agreeing to participate in the trial were stratified by centre (two strata: Manchester and Nottingham) and the size of the patient population in each general practice (three strata: <2500, 2500–6000, >6000) and randomly allocated within strata (1:1 ratio) to the two treatment arms.

The reason for stratifying by centre was to help ensure an even distribution of practices allocated to each of the intervention groups within each centre. The reason for stratifying by size of the patient population in each general practice was because a trial of educational outreach suggested that the larger the practice the more difficult it is to make changes[24].

Block randomisation, using non-predictable block sizes of either two or four, was used to ensure a similar number of practices in each arm. Practices were centrally randomised as they were recruited using the independent web-based randomisation service provided by the Clinical Trials Unit (CTU) at the University of Nottingham. Access to the sequence was confined to the CTU Data Manager (who was independent from the study team). The sequence of treatment allocations will remain concealed until all data analyses have been completed.

### Outcome measures

Outcome measures are measured at the following two time points:

• Six months after the end of the intervention period

• 12 months after the end of the intervention period.

A summary of the main outcome measures is shown Table 1. The measures are described in more detail below.

**Table 1:** Summary of main outcome measures used in the trial

Outcome measure number	Brief description of outcome measure
1	Patients with a history of peptic ulcerwho have been prescribed a non-selective NSAID

2	Patients with asthmawho have been prescribed a beta-blocker

3	Patients aged 75 years and older who have been prescribed an ACE inhibitor or a loop diuretic long-term who have not had a computer-recorded check of their renal function and electrolytes in the previous 15 months

4	Proportions of women with a past medical history of venous or arterial thrombosis who have been prescribed the combined oral contraceptive pill

5	Patients receiving methotrexate for at least three months who have not had a recorded full blood count and/or liver function test within the previous three months

6	Patients receiving warfarin for at least three months who have not had a recorded check of their INR within the previous 12 weeks

7	Patients receiving lithium for at least three months who have not had a recorded check of their lithium levels within the previous three months

8	Patients receiving amiodarone for at least six months who have not had a thyroid function test within the previous six months

9	Patients receiving prescriptions of methotrexate without instructions that the drug should be taken weekly

10	Patients receiving prescriptions of amiodarone for at least one month who are receiving a dose of more than 200 mg per day

#### Primary outcome measures

We are using the following primary outcome measures based on proportions of:

1. Patients with a history of **peptic ulcer **who have been prescribed a non-selective **NSAID**:

• More specifically, those with a computer-coded diagnosis of peptic ulcer disease, at least six months prior to data collection, who have a computer record for one or more prescriptions for a non-selective NSAID in the six months prior to data collection who have not also had a prescription for a PPI within that six month period

• It should be noted that the denominator for this outcome measure is patients with a computer-coded diagnosis of peptic ulcer disease, at least six months prior to data collection, who have not also had a prescription for a PPI in the six months prior to data collection.

2. Patients with **asthma **who have been prescribed a **beta-blocker**:

• More specifically those with a computer-coded diagnosis of asthma, at least six months prior to data collection, who have a computer record of one or more prescriptions for a beta-blocker (oral preparations or eye drops) in the six months prior to data collection

• The denominator for this outcome measure is patients with a computer-coded diagnosis of asthma, at least six months prior to data collection.

3. Patients **aged 75 years and older **who have been prescribed an **ACE inhibitor or a loop diuretic **long-term (see below) who have not had a computer-recorded check of their renal function and electrolytes in the previous 15 months:

• More specifically, long-term prescribing implies a first prescription for an ACE inhibitor or a loop diuretic at least 15 months before the time of data collection and at least one prescription in the six months beforehand

• The denominator for this outcome measure is patients aged 75 years and older who have been prescribed an ACE inhibitor or a loop diuretic long-term according to the above definition.

#### Secondary outcome measures

We are collecting data on a number of secondary outcome measures relating to contraindicated prescribing, inadequate monitoring and dosing problems.

#### Contraindicated prescribing

4. Proportions of women with a past medical history of **venous or arterial thrombosis **who have been prescribed the **combined oral contraceptive pill**

• More specifically, women with a history of venous or arterial thrombosis recorded at least six months prior to data collection who have a computer-recorded prescription for the combined oral contraceptive pill in the six months prior to data collection.

#### Inadequate monitoring

These outcomes are based on proportions of:

5. Patients receiving **methotrexate **for at least three months who have not had a recorded full blood count and/or liver function test within the previous three months

• More specifically

5a: patients with one or more prescriptions for methotrexate recorded on computer three to six months prior to data collection *and *in the three months prior to data collection who have not had a computer-recorded full blood count within the previous three months

5b: patients with one or more prescriptions for methotrexate recorded on computer three to six months prior to data collection *and *in the three months prior to data collection who have not had a computer-recorded liver function test within the previous three months.

6. Patients receiving **warfarin **for at least three months who have not had a recorded check of their INR within the previous 12 weeks

• More specifically, patients with one or more prescriptions for warfarin recorded on computer three to six months prior to data collection *and *in the three months prior to data collection who have not had a computer-recorded INR within the previous three months.

7. Patients receiving **lithium **for at least three months who have not had a recorded check of their lithium levels within the previous three months

• More specifically, patients with one or more prescriptions for lithium recorded on computer three to six months prior to data collection *and *in the three months prior to data collection who have not had a computer-recorded lithium level within the previous three months.

8. Patients receiving **amiodarone **for at least six months who have not had a thyroid function test within the previous six months

• More specifically, patients with one or more prescriptions for amiodarone recorded on computer 6–12 months prior to data collection and in the three months prior to data collection who have not had a computer-recorded thyroid function test within the previous six months.

#### Dosing problems

These outcomes are based on proportions of:

9. Patients receiving prescriptions of **methotrexate **without instructions that the drug should be taken **weekly**.

• More specifically, patients with one or more prescriptions for methotrexate recorded on computer within the three months prior to data collection who do not have the term "weekly" or "week" in the dosage instructions field of the latest prescription for the drug.

10. Patients receiving prescriptions of **amiodarone **for at least one month who are receiving a dose of more than 200 mg per day

• More specifically, patients with evidence of being prescribed amiodarone 200 mg tablets for more than one month in the three months prior to data collection, who do not have the term "once daily" (or similar) in the dosage instructions field for the drug.

#### Additional outcome measure relating to prescription of beta-blockers to patients with asthma

This secondary outcome measure is based on proportions of patients with **asthma **who do **not **have coronary heart disease (CHD) and have been prescribed a **beta-blocker**:

• More specifically those with a computer-coded diagnosis of asthma and no record of CHD, at least six months prior to data collection, who have a computer record of one or more prescriptions for a beta-blocker (oral preparations or eye drops) in the six months prior to data collection

• The denominator for this outcome measure is patients with a computer-coded diagnosis of asthma and no computer-coded record of CHD, at least six months prior to data collection.

#### Composite outcome measures

We will also use data from the above outcome measures to create a series of composite outcome measures (see analysis section for further details).

### Ascertainment of outcomes

#### Clinical outcomes

During the first three months of the study we worked with the company that produce Quest Browser software  to develop computerised queries that would produce precisely the same types of data as we have used in our pilot study of primary outcome measures that used QRESEARCH practices . We also worked with the company to produce the outputs needed for the secondary outcome measures.

For each practice agreeing to be involved in the trial, Quest Browser software was installed on their clinical computer system. At the time of installation of the software, a search of the GP computer system, using Quest Browser was undertaken to provide anonymised baseline data and details of individual patients at risk from potentially hazardous prescribing and medicines management.

Anonymised and encrypted data pertaining to the computerised primary and secondary outcomes measures are sent via the Internet to a secure computer at the University of Nottingham. Using Quest Browser Central software the anonymised data are automatically imported in an Access database along with a unique code identifying the practice.

Further data are collected at six and 12 months after the completion of the 12-week intervention period in practices in each arm of the trial.

#### Issues concerning ascertainment of secondary clinical outcome measures

Over the course of the study we have identified issues that may limit the validity of three of our secondary outcome measures.

As already noted, for outcome number six, we have identified seven practices that keep their records of INR results separate from their main practice computer system and thus appear to have very high proportions of patients not having INRs checked according to the computer searches we have used in our study. We will need to exclude these practices from the analysis of this outcome measure.

For outcome number nine, during the course of our study, the NPSA required all GP computer systems to introduce methods of ensuring that electronic prescriptions for methotrexate gave instructions that the medication should be taken weekly[25]. Since this change was introduced our computer searches have been unable to capture the text used to confirm the dosage instructions. Unless we can resolve this problem it is unlikely that we will be able to report on the follow-up data for this outcome measure.

For outcomes nine and 10, we have found that for the eleven practices that use the TPP computer system, we are unable to extract information on dosage instructions. This means that we will not be able to report on these outcome measures for the practices in our trial that use this system.

#### Data for the economic analysis

##### Costs of the intervention

The costs measured at practice level are the costs of setting-up and delivering the intervention. The costs measured at the patient level are any costs of delivering the intervention that can be linked to individual patients. Resources associated with providing the intervention have been recorded, and will be combined with unit costs to produce total costs.

##### Modelling economic analysis

Patients identified from the baseline computer system searches as being at risk of potentially hazardous prescribing and medicines will be included in the modelling economic analysis provided that they give informed consent for researchers to view their records.

Wherever possible, data are extracted electronically, although in the case of correspondence regarding hospital contacts it is usually necessary to anonymise and photocopy relevant information. Anonymised data are sent to the University of Nottingham where data processing and analysis will take place.

Patients in both intervention arms will be followed up for 12 months following the completion of the intervention in each practice. Error-related resource use data will be collected from these patients. NHS resource use data will be collected retrospectively for the 12 month period before the intervention (baseline) and for 12 months after the intervention (follow-up). Unit costs associated with the intervention will be obtained from Personal and Social Services Research Unit , Department of Health  and other reference costs.

#### Data for the qualitative study

Data for the qualitative study have been obtained from:

• 12 initial brief interviews with NHS staff

• 20 in-depth interviews with NHS staff

• Interviews with the six trial intervention pharmacists

• Focus groups involving a total of 30 participants (NHS staff and trial intervention pharmacists)

• Diary records kept by the six trial intervention pharmacists of their activities and experiences

• Interviews with two members of the research team closely involved in roll-out of the trial.

Data generation continued until no new themes or issues were emerging (saturation).

Interviews and focus groups have been digitally recorded and transcribed and accompanying field notes have been retained.

The specific research questions explored in the qualitative study include the following:

• What are the views of primary care staff and pharmacists about the acceptability, effectiveness and long-term sustainability of the trial interventions?

• What barriers, if any, were experienced by trial pharmacists and primary care staff in identifying patients at risk from potentially hazardous prescribing, in correcting these problems once identified, and instituting safer medicines management culture and policies in the pharmacist-led IT-based intervention practice arm?

• What barriers, if any, were experienced by primary care staff in identifying patients at risk from potentially hazardous prescribing, in correcting these problems once identified, and instituting safer medicines management culture and policies in the simple feedback practice arm?

• In which ways might the trial interventions need to be modified or adapted in order to maximise their effectiveness when implemented in routine general practice?

• What alternative interventions might be both acceptable to key stakeholders and effective in reducing potentially hazardous prescribing in general practice?

### Adverse events

A protocol for dealing with serious adverse events that might occur in study practices in patients identified by the PINCER trial outcome measures is shown in Appendix 1 (see additional file 1).

### Sample size

Our sample size calculations are based on the assumption that for the proportion of patients fulfilling the criteria for any one of our primary outcome measures, there will be a maximum 11% reduction in the simple feedback arm and a 50% reduction in the pharmacist intervention arm.

Separate sample size calculations were performed for each of the primary outcome measures (see Table 2). Sample sizes unadjusted for clustering were calculated using the software package nQuery Advisor^® ^version 6.0. [26]

**Table 2:** Sample size calculations for the three primary outcome measures assuming an 11% reduction in error rates for the simple feedback group and a 50% reduction in error rates for the intervention group

Outcome measure	Patients with a history of peptic ulcer who have been prescribed a non-selective NSAID	Patients with asthma who have been prescribed a beta-blocker	Patients aged 75 years and older prescribed an ACE inhibitor or a loop diuretic long-term without a check of their renal function and electrolytes in the previous 15 months
Median error rate^1^ (Interquartile range)	5.76% (3.76% – 7.85%)	1.90% (1.27% – 3.08%)	19.80% (15.13% – 32.69%)

Error rate in control group (assuming 11% reduction)	5.13%	1.69%	17.62%

Error rate in intervention group (assuming 50% reduction)	2.88%	0.95%	9.90%

Intraclass Correlation Coefficient (ICC)^1^	0.01082	0.00657	0.00952

Cluster size^1^	63	439	105

Inflation factor	1.7	3.9	2.0

Total number of practices required	64	66	12

^1 ^Estimated using data obtained from 43 general practices contributing to the QResearch database

Data from 43 general practices contributing anonymous clinical data to the QRESEARCH research database  were used to describe prevalence rates of asthma and peptic ulcer disease and to estimate the median proportions for each of our primary outcome measures. The intracluster correlation coefficients (ICCs) used in the calculation of the design effect (to inflate the sample sizes to adjust for the cluster design)[27] were as follows:

• 0.01082 for patients with a history of peptic ulcer who have been prescribed a non-selective NSAID (excluding those that were also in receipt of PPIs, which would protect against the risks from NSAIDs);

• 0.010657 for patients with asthma who have been prescribed a beta-blocker;

• 0.00952 for patients aged 75 years and older who have been prescribed an ACE inhibitor or a loop diuretic long-term who have not had a computer-recorded check of their renal function and electrolytes in the previous 15 months.

The suggested 11% reduction in the simple feedback arm is the equivalent to the 75% centile for changes observed as a result of audit and feedback in a Cochrane systematic review available at the time that we did our sample size calculations. [28]

The suggested 50% reduction in the pharmacist intervention arm of the trial is based on extrapolation from our pilot studies[18,19] and findings from systematic reviews and other studies that, at the time of applying for funding for our study, showed that:

• Pharmacist-led interventions can lead to resolution of medication-related problems in 55–93% of patients [29-33]

• Educational outreach is a moderately powerful tool for changing professional behaviour[34]

• Multifaceted interventions aimed at different barriers to change are more effective than single interventions[35].

The calculation shown in Table 2 indicates that we would need at least 66 practices to detect a difference between an 11% reduction in error rate in the simple feedback arm and a 50% reduction in the intervention arm for each of our three primary outcome measures.

On the basis of these calculations, we decided to aim to recruit at least 68 practices. With 34 practices in each of the two treatment arms, we would have at least 80% power (two-tailed alpha of 0.05) to demonstrate a 50% reduction in rates of potentially hazardous prescribing and medicines management in the pharmacist-led arm compared with 11% in the simple feedback arm.

### Compliance

We recognise that it can be a challenge to encourage general practices to engage in interventions. However, as the intervention involves either simple feedback, or feedback and the provision of a pharmacist to work with the practices, we do not expect non-compliance with the intervention to be a large problem. In addition, from our experience of the pilot study and of conducting previous trials, we believe that the risks of non-compliance will be minimised by providing practices with clear information on what the study involves, providing access to members of the research team to answer queries and address problems experienced by the practices, and support from the PCTs.

### Likely rate of loss to follow up

We do not envisage practices dropping out of the study once they have agreed to take part. Nevertheless, at the outset we have stressed to the practices the importance of allowing us to collect follow-up data, even if the practice has not engaged fully in one of the interventions. As outcome data collection will require minimal input from the practice or its staff, we do not foresee problems with obtaining outcome data from participating practices.

Some patients will move practices and some will die within the intervention period. However, this is unlikely to have a large impact on the proportion of patients with errors at follow-up, unless leaving the practice or death is differentially related to medication error. This is unlikely because the number of deaths attributable to the medication errors we are studying is likely to be small during the course of the study. Nevertheless, we will try to follow up patients who have died by viewing their electronic medical records up until the time of death, and requesting paper-based records that might contain details of contacts with secondary care.

### Withdrawal of patients from the study

Withdrawal of patients consenting for their medical records to be examined will not affect the analysis of clinical outcomes as these are obtained from anonymised computer searches of all "at risk" patients in the general practices recruited to the study.

Withdrawal of these patients will affect the health economic analysis although it is expected that numbers of patients withdrawing will be small. A patient will be considered to have withdrawn from the study if the study team receive any notification that the patient wishes to withdraw. This notification might come from the patient themselves, the patient's representative or from the patient's general practice.

### Statistical analysis

Data analysis, using the following analysis plan, will be undertaken blind to treatment arm allocation (i.e. the treatments will be identified only as A and B until analysis is complete). The primary analysis for the clinical outcomes used in the trial will be undertaken using the six-month follow-up data.

### Descriptive analyses

Continuous data will be explored using frequencies and histograms and described using means and standard deviations (SD) if approximately normally distributed and medians and inter-quartile ranges (IQR) if non-normally distributed. Categorical data will be described using frequencies and percentages. Characteristics of participating practices will be compared informally between treatment arms.

#### Describing baseline characteristics of patients and practices

a) The following characteristics will be described by treatment arm:

i) Patient age and gender

ii) Practice list size (median and IQR, or mean and SD if normally distributed)

iii) Practice population by age group (number and %)

iv) Practice deprivation using the Index of Multiple Deprivation (IMD) 2004[36] (median and IQR, or mean and SD if normally distributed) Note this has been calculated by multiplying the proportion of the total list size living in each Lower Layer Super Output Area (LSOA) by IMD 2004 LSOA level score and then summing these across all LSOAs in which patients registered at the practice live.

v) Practice training status (%)

vi) Practice Quality and Outcomes Framework (QOF) medicines management indicator points and total QOF points[37] if available (Mean (SD or median (IQR) dependent on distributions).

#### Describing baseline prevalence of medication-related problems

The following will be described using the numerator, denominator and percentage by treatment arm, at patient level:

i) Primary outcome measures:

Patients with a history of peptic ulcer prescribed an NSAID without a PPI (numerator)/

Patients with a history of peptic ulcer without a PPI (denominator)

Patients with asthma prescribed a beta-blocker (numerator)/Patients with asthma (denominator)

Patients aged ≥75 on long term ACE inhibitors or diuretics without urea and electrolyte monitoring in the previous 15 months (numerator)/

Patients aged ≥75 on long term ACE inhibitors or diuretics (denominator)

ii) Secondary outcome measures:

Patients with asthma and not CHD prescribed a beta-blocker (numerator)/

Patients with asthma and not CHD (denominator)

Female patients with a history of venous or arterial thromboembolism and arterial thrombosis prescribed combined oral contraceptives (numerator)/

Female patients with a history of venous or arterial thromboembolism and arterial thrombosis (denominator)

Patients prescribed methotrexate for ≥ three months without a full blood count in last three months (numerator)/

Patients prescribed methotrexate for ≥ three months (denominator)

Patients prescribed methotrexate for ≥ three months without an liver function test in last three months (numerator)/

Patients prescribed methotrexate for ≥ three months (denominator)

Patients prescribed warfarin for ≥ three months without an INR in last three months (numerator)/

Patients prescribed warfarin for ≥ three months (denominator)

Patients prescribed lithium for ≥ three months without a lithium level in last three months (numerator)/

Patients prescribed lithium for ≥ three months (denominator)

Patients prescribed amiodarone for ≥ six months without a thyroid function test in the last six months (numerator)/

Patients prescribed amiodarone for ≥ three months (denominator)

Patients prescribed methotrexate without instructions to take weekly (numerator)/

Patients prescribed methotrexate (denominator)

Patients prescribed amiodarone for ≥ one month at a dose >200 mg/day (numerator)/

Patients prescribed amiodarone for ≥ one month (denominator)

Four composite outcome measures will also be used comprising:

Number of patients with at least one prescribing problem (numerator)/

Number of patients at risk of at least one prescribing problem (denominator)

Number of patients with at least one monitoring problem (numerator)/

Number of patients at risk of at least one monitoring problem (denominator)

Number of patients with at least two prescribing problems (numerator)/

Number of patients at risk of at least two prescribing problems (denominator)

Number of patients with at least two monitoring problems/

Number of patients at risk of at least two monitoring problems (denominator)

#### Describing outcome data

The prevalence of each primary and secondary outcome measure listed above will be described using numerators, denominators and percentage by treatment arm separately at six and 12 months follow up.

### Comparing baseline characteristics between treatment arms

Baseline characteristics will be compared informally between treatment arms[38].

### Other baseline analyses

We may undertake other baseline analyses, and if so these will be described in a separate analysis plan.

### Comparisons between treatment arms

All outcome measures are binary in nature. They will be compared between treatment arms using random effects logistic regression with patient at level one and practice at level two. Odds ratios and 95% confidence intervals will be estimated using two-level random intercepts logistic models, with patients at level one and practices at level two. Models will include randomisation stratum as a fixed effect[39]. Three separate analyses will be undertaken [40-42]:

i) adjusting only for stratum (practice level)

ii) adjusting for stratum (practice level) and for the presence of medication-related problems at baseline (patient level).

iii) adjusting for stratum (practice level), baseline medication-related problems (patient level), and deprivation and training status (practice level).

An intention-to-treat analysis will be used such that practices will be analysed in the arms they were allocated to regardless of whether they received the intervention or not[43,44]. Significance will be assessed based on likelihood ratio tests with a p value of < 0.05 taken as significant. Analyses will be undertaken using Stata version 10[45]. Regression models adjusted for stratum and baseline medication problem rates will yield estimates of the cluster and residual variance components, enabling the ICCs to be calculated [45].

#### Primary outcomes

The proportions of "at risk" patients in each treatment arm with the errors of interest will be compared between treatment arms at six and 12 months after the end of the intervention period in each practice.

The ICC and 95% confidence interval will be estimated for each of the primary outcome measures from the regression models adjusted for stratum and baseline medication errors.

If any practices are lost to follow up, a sensitivity analysis will be undertaken replacing the missing follow up data with the baseline data for that practice[43,44].

It should be noted that some patients (for both primary and secondary outcome measures) will become "at risk" between the time of the baseline data collection and the follow up data collections. For the primary outcome measures this may occur because:

• They have a diagnosis of asthma or peptic ulcer recorded on the computer following the time of the baseline data collection

• They reach the age of 75 years following the time of the baseline data collection

• At time of the baseline data collection, they do not fall within our definition of being prescribed an ACE inhibitor or a loop diuretic long-term, but they do so at the six and/or 12 month follow up data collection points

• They join the practice after the time of baseline data collection and fall within one of the "at risk" groups.

We believe that it is important to include these patients in the analysis because the intervention is aimed not only at correcting hazardous prescribing, but also at introducing systems to prevent future errors. Nevertheless, for the primary outcome measures, sensitivity analysis will be undertaken excluding patients that (for the reasons outlined above) become "at risk" between the baseline data collection and the follow up data collections.

#### Secondary outcome measures

The proportion of "at risk" patients in each treatment arm with the error of interest will be compared between treatment arms at six and 12 months after the end of the intervention period.

We acknowledge the potential for type 1 errors associated with significance testing for multiple end points. We will therefore consider our analyses of secondary outcome measures to be partly exploratory in nature, and partly confirmatory of our findings for the primary outcome measures.

### Model checking

Level two residuals (variation between practices) for each model will be assessed for normality and constant variance by normal plots and plotting residuals against fitted values. The variation between practices will be summarised by treatment arm[46].

### Multiple endpoints

No adjustments will be made for multiple endpoints. Findings will be interpreted with caution in view of the number of statistical tests undertaken.

### Sub-group analyses

Sub-group analyses[38] will be undertaken only for primary outcome measures. The following analyses will be undertaken:

i) assessing whether the effect of the intervention varies by practice size

ii) assessing whether the effect of the intervention varies by practice deprivation

These analyses will be undertaken by incorporating a term for the interaction between treatment arm and the (continuous) covariate of interest into the regression model[38]. Where there is evidence of non-linearity the covariate will be categorised at the median value. Significance will be assessed based on likelihood ratio tests with a p value of < 0.05 taken as significant and p values between 0.05 and 0.1 described as there being "some evidence" for an interaction.

### Missing data

A complete case analysis will be undertaken at six and 12 months. If any practices are lost to follow up we will consider, prior to unblinding those undertaking the analysis to treatment group allocation, a range of approaches for undertaking sensitivity analyses to assess the robustness of the findings with respect to missing data.

### Comparing characteristics of participating and non-participating practices

The following characteristics will be compared between participating and non-participating practices:

i) List size (using t test or Mann Whitney U test dependent on distribution)

ii) Number of GPs (Mann Whitney U test)

iii) % of practice population aged ≥ 75 years (using t test or Mann Whitney U test dependent on distribution)

iv) Training status (using χ^2 ^test)

v) Deprivation using IMD2004 (using t test or Mann Whitney U test dependent on distribution).

#### Economic analysis

We will undertake a two-stage economic analysis:

• A within trial analysis of cost per error avoided;

• A modelling analysis of economic impact of error reduction.

A standard approach to economic analysis will be applied[47].

The principal objective of the within-trial analysis is to identify and value the resource use associated with the interventions used in the trial.

The principal objectives of the modelling analysis are to:

• Identify and value the impact on patients' health status of the interventions;

• Identify and value the resource use associated with reduced prescribing errors in primary care;

• Assess the relative value for money of the interventions.

### Perspective

We propose to undertake the economic analyses from the perspective of the NHS in terms of the direct costs of providing an intervention to reduce prescribing errors in general practice.

### Comparators and key parameters under investigation

**Figure 1:**
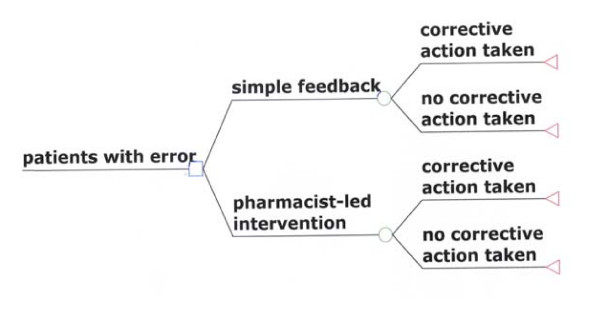
**decision-analytic model of intervention to reduce preventable drug related morbidity arising from prescribing errors in general practice**.

The evaluation will compare the pharmacist-led intervention with simple feedback. Figure 1 illustrates the comparators and the probabilistic events that are associated with each strategy.

For the purposes of the within trial analysis of cost per error avoided we will examine the differences in costs of the pharmacist-based intervention compared with simple feedback, in the context of error rate reduction.

For the modelling analysis of cost per quality adjusted life year (QALY) gained, we will examine the differences in overall NHS costs, in the context of QALYs gained.

### Sample size for the economic analysis

The study cannot be powered to detect differences in costs because there is no prior study upon which to base a power calculation. From the 72 practices recruited to the trial, just over 1000 patients (who have been identified to be at risk from potentially hazardous prescribing or medicines management) have consented for the research team to access their medical records for the purposes of the economic analysis.

### Outcome measures to be included in the economic analysis

Pilot work on data collection for the economic analysis showed this to be very time consuming and we therefore decided it would not be possible to collect data for all the outcome measures. We will collect data on outcome measures 1, 2, 3, 5, 7 and 8. We have thus decided not to collect data for the economic analysis for outcome measures 6, 9 and 10, partly because of the problems we have had with data ascertainment (as outlined above). In addition, the numbers of patients identified for outcome measure four are too low to allow for meaningful within-trial economic analysis and so this outcome measure will also not be included.

### Time horizon (follow-up period)

Error rates in practices in both groups will be followed up for 12 months following the completion of the intervention in each practice

### Costs

In this cluster trial, costs will be incurred at both the patient and cluster (practice) level. Randomising by cluster can lead to imbalances between treatment arms in practice or patient level factors, including resource use.

#### Costs measured at practice level

The costs measured at practice level will be the costs of setting-up and delivering the intervention.

#### Costs measured at patient level

The costs measured at the patient level will be any costs of delivering the intervention that can be linked to individual patients.

In the modelling analysis, error-related resource use data will be collected from patients who have consented to access to their medical records ("consented numerator"). NHS resource use data will be collected retrospectively for the 12 month period before the intervention (baseline) and for 12 months after the intervention (follow-up) for the consented numerator. This will allow us to adjust our analyses for baseline cost[48].

Variable costs refer to items where the quantity of resources used is determined only by the need for them as inputs to individual patient care. Variable resource use associated with the interventions (time spent, costs of telephone calls, printing and posting) will recorded for each patient. Fixed costs are those costs that are not affected by patient activity in the short term. UK standard costs will be used for unit costs. This may somewhat over- or under-estimate local unit costs, but allows explicit comparison of costs and local adjustments can be made. Unit costs associated with the intervention will be obtained from the Personal Social Services research Unit (PSSRU)[49], Department of Health reference tables and other reference costs.

### Within trial analysis of outcomes

Outcomes used in the within-trial analysis of cost per error avoided will be number of prescribing errors in each treatment arm.

### Modelling analysis of outcomes

Outcomes used in the modelling analysis will be derived from published evidence on the link between error reduction and impact on health. The PINCER study is not designed to calculate the impact of the intervention on patient health outcomes, either in terms of sample size or length of follow-up. Use of proxy measures such as number of primary and secondary care contacts (hospital admissions, accident and emergency visits and outpatient visits) may be subject to difficulties if considered as patient outcomes. This is because the intervention may lead to increased NHS contact in the short term. We will model the long-term benefit associated with avoidance of errors.

Literature review will be used to obtain published utility weights to allow QALY generation and cost utility analysis.

### Within trial analysis of costs

Costs calculated in the within-trial analysis will be cost of intervention for each patient enrolled in the trial for both treatment arms. These data will be presented separately for the two treatment arms. If required, differences in the mean cost between the treatment arms will be estimated using a two level random linear effects model with patient at level one and practice at level two, to take account of the clustered nature of the data. Baseline costs at practice level will be included as covariates in the model. The assumptions for undertaking random effects linear regression will be assessed using residual plots and in addition the robustness of the analysis to non-normality will be assessed by estimating 95% confidence intervals around the difference in mean costs using bias corrected non-parametric bootstrapping. If the assumptions for undertaking random effects linear regression are not met, we will consider aggregating the patient level cost data to the level of the practice. Comparisons between treatment arms at practice level will be made using a two sample t-test on the original dataset, or on a bootstrapped dataset, depending on the normality of the distribution of practice level costs[50].

### Modelling analysis of costs

The practice level cost of the intervention will be combined with patient-level costs of error management. The cost per patient will be estimated over the study period. The costs for each event (GP surgery visit, investigations, out of hours, accident and emergency, outpatient and hospital admission costs) will be estimated for each patient in the trial for both treatment arms. The costs will be calculated as resource use multiplied by unit cost and will be reported descriptively, both as resource use and cost data, for each error, with means and ranges presented.

These data will be used to populate the modelling economic analysis. Where there are gaps, these data will be supplemented with data from the literature. Distributional forms of secondary data will follow modelling convention. Literature review will be used to validate the primary error-related resource use data and to assess any long term resource use consequences not detected during the study period.

### Incremental cost-effectiveness ratios (ICERs)

Incremental cost effectiveness ratios will be calculated for the trial-based analyses and sensitivity analyses.

The effectiveness measure for the calculation of incremental cost effectiveness ratio will be cost per unit of outcome. For the within-trial analysis we will use errors. If the lower cost intervention is also associated with better outcomes than the more costly comparator, this will be treated as efficient. In this scenario, incremental ratios would not be calculated for this intervention, since its use would lead to both net savings and greater benefits. Incremental cost effectiveness ratios will be calculated if the higher cost intervention is associated with better outcomes. The incremental ratios will be calculated as:



Statistical analysis is not appropriate to test the robustness of ICERs. It is not possible to generate 95% confidence intervals around ICERs because the ratio of two distributions does not necessarily have a finite mean, or therefore, a finite variance[50]. Therefore, generation of a bootstrap estimate of the ICER sampling distribution to identify the magnitude of uncertainty around the ICERs is required. Bootstrapping with replacement will be employed, utilising MS Excel^®^, using a minimum of 1000 iterations to obtain 2.5% and 97.5% percentiles of the ICER distribution.

### Cost effectiveness acceptability curves and net benefit

A cost effectiveness acceptability curve will be constructed to express the probability that the cost per extra unit of outcome gained from within the trial (y-axis) is cost effective as a function of the decision-maker's ceiling cost effectiveness ratio (λ) (x-axis)[51]. Net benefit will be determined when λ is £30 000. For the within-trial analysis we will use cost per error avoided.

### Sensitivity analysis

Sensitivity analysis is required to assess the level of uncertainty in the data collected within the trial and subsequent internal robustness of the results.

The key aspect to be investigated in this analysis will be the impact of:

• size of practice

• alternative models of service delivery.

#### Format of tables for publishing the main trial results and within trial economic analysis

The format of tables for publishing the main trial results and within trial economic analysis is shown in Appendix 2 (see additional file 2).

#### Qualitative study

The qualitative data will be subjected to thematic analysis with the aid of NVivo^® ^software. Actively searching for disconfirming data and regular detailed discussions amongst the qualitative sub-group, and periodic discussions with the wider multi-disciplinary steering group will help ensure the rigour of the analysis.

#### Trial organisation

Professor Avery will have overall responsibility for the day-to-day management of the trial and for the conduct of the trial in the area around Nottingham. Professor Avery will head the Trial Management Group.

Professor Cantrill will have overall responsibility for the conduct of the trial in the area around Manchester.

Professor Elliott will have responsibility for the economic analysis.

Professor Sheikh will have overall responsibility for the conduct of the qualitative analysis and statistical advice, which will be run from the University of Edinburgh.

Dr Sarah Armstrong is the trial statistician.

### Trial Management Group

A Trial Management Group has met of a quarterly basis throughout the study to help ensure that all trial activities are organised according to the protocol and within the timescales set out in the original application for funding.

### Trial Steering Committee

The Trial Steering Committee (TSC) will monitor and supervise the trial and comment on any proposed amendments' to the protocol. The Trial Steering Committee is headed by Professor Philip Hannaford. Professor Martin Buxton and Dr Marjorie Weiss are the other external members of the committee. The TSC and has agreed to operate within the framework suggested in the *MRC Guidelines for good clinical practice in clinical trials *[15].

### Data Monitoring and Ethics Committee (DMEC)

The DMEC is headed by Professor Richard Baker. Other external members of the committee are Professor Christine Bond and Professor Peter Donnan. The trial statistician will report to the DMEC, which will be responsible for reviewing the data from the trial. The DMEC has agreed to operate within the framework suggested in the *MRC Guidelines for good clinical practice in clinical trials *[15].

#### Ethical aspects

The clinical trial will be conducted according to the Helsinki Declaration[52], the Good Clinical Practice Guidelines[53] and NHS Research Governance requirements.

Patients agreeing for the study team to access their clinical records have provided written informed consent in a form designed for such purpose. The patient may refuse to continue participating in the study at any time after providing his/her consent.

The information generated by the study will be confidential and limited to the purposes stipulated in the protocol.

The study has been approved by Nottingham 2 Research Ethics Committee (Reference: 05/Q2404/26). All staff involved in data collection will have approval from the appropriate local NHS research and development offices.

#### Study timeline

Trial start: 1 April 2006

Start of baseline data collection and interventions in general practices: August 2006

End of interventions in general practices: February 2008

End of 12 month follow-up data collection: April 2009

Start of data analysis: May 2009

Planned study end date: 30 September 2009

Duration: 3.5 years

## Discussion

At the time of submission of this article, 72 general practices had been recruited (36 in each arm of the trial) and the interventions had been completed.

In keeping with findings from our pilot studies, we have been able to recruit general practices without too much difficulty. It is likely that our success in recruitment relates to the attention to detail we put into developing a feasible intervention that was seen to be relevant to general practices, although we will find out more about whether this was the case from our qualitative study. In addition, we put considerable effort into our recruitment strategy including pre-publicising the trial, sending documentation to general practices about the trial and following up on this to arrange visits to practices where the study was explained in more detail.

Baseline data extraction was undertaken smoothly and successfully and provided the information needed for practices to consider acting on patients with potentially hazardous prescribing and medicines management. In particular, data extraction for our pre-specified primary outcome measures appears to have worked successfully in all general practices recruited to the study. Nevertheless, we have encountered some difficulties with the secondary outcome measures, as described above. Some of these problems could have been avoided with better foresight on behalf of the research team. For example, had we been aware that some general practices kept a separate record of INR results, and did not keep these on their main clinical computer systems, we could have excluded such practices from our recruitment. Similarly, if we had realised that it would not be possible to extract dosage instructions for general practices using the TPP computer system, we could have excluded practices using this computer system (we did pilot our outcome measures using the TPP computer system and went ahead with recruiting practices because we erroneously believed that was a solution to the problem). For outcome measure nine, which focused on dosage instructions for methotrexate, a national policy change that occurred during the course of our study has meant that we will be unable to collect useful follow-up data on this outcome measure for any of the practices we have recruited. This is because the introduction of a forcing function to GP computer systems, which does not allow for anything but weekly dosing, does not appear as a line of dosing text that can be picked up by MIQUEST software. It is unfortunate that our study will not be able to demonstrate the impact of this important safety initiative, although it is likely that the introduction of this computerised safety feature will have brought about improvements in both arms of the trial.

At the time of submitting this protocol, analysis of quantitative data had not been undertaken.

## Abbreviations

ACE: Angiotensin converting enzyme (inhibitor); CHD: Coronary heart disease; CTU: Clinical Trials Unit; DMEC: Data Monitoring and Ethics Committee; GP: General practitioner (or family practitioner); ICC: Intraclass correlation coefficient; ICER: Incremental cost effectiveness ratios; INR: International normalised ratio; IT: Information technology; MRC: Medical Research Council; NHS: The UK National Health Service; NPSA: National Patient Safety Agency; NSAIDs: non-steroidal anti-inflammatory drugs; PCT: Primary Care Trust; PPI: proton pump inhibitor; TPP: The Phoenix Partnership (the name of a GP computer system); TSC: Trial Steering Committee.

## Competing interests

The authors declare that they have no competing interests.

## Authors' contributions

AJA, who has made substantial contributions to the conception and design of the study, is co-responsible for the overall administration and direction of the project, the analysis and interpretation of data and will give the final approval of the version to be published. JAC and AS are also co-responsible for the overall design, administration and direction of the study. SR is the Trial Co-ordinator and is responsible for the day-to-day management of the trial. She was involved in the design of the Quest Browser queries and the piloting of the data extraction methods. JAC, AS, SA, RE, RH, DK, CJM, SM, RJP and KC also participated in the design of the project: AS, SA, DK and RJP have had a major role in designing the statistical analysis for the trial; RE has led on the design of the economic analysis; AS, SM and KC have led on the design of the qualitative analysis.

## Funding

Patient Safety Research Program of the UK Department of Health.

## Supplementary Material

Additional file 1 **Pincer Protocol for Publication Appendix 1**: Protocol for dealing with serious adverse events in the PINCER trial Click here for file

Additional file 2 **Pincer Protocol for Publication Appendix 2**: Format of tables for publishing the main trial results and within trial economic analysis Click here for file
