# Correction: Protocol for the PINCER trial: a cluster randomised trial comparing the effectiveness of a pharmacist-led IT-based intervention with simple feedback in reducing rates of clinically important errors in medicines management in general practices

**DOI:** 10.1186/1745-6215-11-23

**Published:** 2010-03-02

**Authors:** Anthony J Avery, Sarah Rodgers, Judith A Cantrill, Sarah Armstrong, Rachel Elliott, Rachel Howard, Denise Kendrick, Caroline J Morris, Scott A Murray, Robin J Prescott, Kathrin Cresswell, Aziz Sheikh

**Affiliations:** 1 Division of Primary Care, The Medical School, Queen's Medical Centre, Nottingham, NG7 2UH, UK; 2 Division for Social Research in Medicines and Health, The School of Pharmacy, University of Nottingham, University Park, Nottingham, NG7 2RD, UK; 3 Drug Usage & Pharmacy Practice Group, School of Pharmacy & Pharmaceutical Sciences, University of Manchester, Oxford Road, Manchester, M13 9PL, UK; 4 Trent Research Design Service, Division of Primary Care, Tower Building, University Park, Nottingham, NG7 2RD, UK; 5 School of Pharmacy, University of Reading, PO Box 226, Whiteknights, Reading, RG6 6AP, UK; 6 Department of Primary Health Care and General Practice, Wellington School of Medicine and Health Sciences, University of Otago, Mein Street, Wellington South, New Zealand; 7 Centre for Population Health Sciences, University of Edinburgh, 20 West Richmond Street, Edinburgh, EH8 9DX, UK

## Correction

Following the publication of our article [1] we noticed an error in the Methods section. In the sub-section 'Describing baseline prevalence of medication-related problems' the denominator for the following secondary outcome measures is incorrectly stated:

Patients prescribed amiodarone for ≥ six months without a thyroid function test in the last six months (numerator)/

Patients prescribed amiodarone for ≥ three months (denominator)

This should read:

Patients prescribed amiodarone for ≥ six months without a thyroid function test in the last six months (numerator)/

Patients prescribed amiodarone for ≥ six months (denominator)
