# Supplementary material for: Protocol for the PINCER trial: a cluster randomised trial comparing the effectiveness of a pharmacist-led IT-based intervention with simple feedback in reducing rates of clinically important errors in medicines management in general practices
Source: Trials. 2009 May 1;10:28. doi: 10.1186/1745-6215-10-28 (PMC2685134; doi:10.1186/1745-6215-10-28)
Supplement: Additional file 1 — Pincer Protocol for Publication Appendix 1: Protocol for dealing with serious adverse events in the PINCER trial [file 1745-6215-10-28-S1.doc]

**APPENDIX 1: PROTOCOL FOR DEALING WITH SERIOUS ADVERSE EVENTS IN THE PINCER TRIAL**

**Background**

Below is a list of serious adverse events that might occur in study practices in patients identified by the PINCER Trial outcome measures.

| **Outcome measure** | **Potential adverse event** |
| --- | --- |
| 1 | Serious GI bleed in a patient with a history of peptic ulcer receiving a non-selective NSAID without PPI cover |
| 2 | Serious asthma attack in a patient with a history of asthma who has been prescribed a beta-blocker |
| 3 | Admission to hospital with a serious electrolyte disturbance or dehydration in a patient aged 75 years and older who has been prescribed an angiotensin converting enzyme inhibitor (ACEI) or a loop diuretic long-term who has not had a recorded check of their renal function and electrolytes in the previous 15 months |
| 4 | Venous or arterial thrombosis in a woman with a past medical history of venous or arterial thrombosis who has been prescribed a combined hormonal contraceptive (CHC) |
| 5 | Serious haematological or liver problem in a patient receiving methotrexate for at least three months who has not had a recorded full blood count and / or liver function test within the previous three months |
| 6 | Serious bleed associated with high INR, or thromboembolic event associated with low INR, in a patient receiving warfarin for at least three months who has not had a recorded check of their international normalised ratio (INR) within the previous 12 weeks |
| 7 | Lithium toxicity in a patient receiving lithium for at least 3 months who has not had a recorded check of their lithium levels within the previous 3 months |
| 8 | Thyrotoxicosis in a patient receiving amiodarone for at least 6 months who has not had a thyroid function test within the previous 6 months |
| 9 | Toxic effects from methotrexate overdose in a patient receiving prescriptions of methotrexate without instructions that the drug should be taken weekly |
| 10 | Toxic effects from amiodarone overdose in a patient receiving prescriptions of amiodarone for at least one month without instructions to take a dose of 200mg or less per day |

Given that the outcome measures are based on identifying patients *at risk* from a serious adverse event, rather than adverse events themselves, the study team will not automatically be made aware of all patients suffering such events.

In some cases we will learn of adverse events, if they occur. For example, we intend to collect information on adverse events for the health economic analysis, but this will take place only for those patients giving prior consent (currently 30-40% of patients are giving consent to be involved in this part of the study). Also, for these patients, data collection will take place at least one year after the intervention has been completed in a particular practice. In general practices receiving pharmacist intervention, it is possible that the pharmacists will come across patients who have suffered a serious adverse event.

Overall, it is clear that we do not have a reliable way of identifying all serious adverse events involving patients identified by the PINCER Trial outcome measures. The main reason for this (as outlined above) is that the trial was designed to investigate changes in proportions of patients *at risk* rather than adverse events themselves. Also, we decided to use anonymised data in order to increase the generalisability of the results and not to adversely affect the relationship between general practices and their patients.

Assuming that it would not be appropriate to alter the study protocol to include obtaining information on individual patients without their consent, we suggest that the protocol for dealing with serious adverse events should relate only to those adverse events that the study team is made aware (e.g. through the study pharmacists or the health economic analysis).

**Protocol**

It is the responsibility of general practices to deal appropriately with serious adverse events involving their patients. This includes:

- Prompt action to deal with the adverse event, which might include referral to hospital
- An explanation to the patient of what has gone wrong and why, including admission of fault if an error has occurred

For serious adverse events that come to the attention of study pharmacists, or the study team (through viewing patients’ records as part of the health economic analysis), we will ensure that information about these events is fed back to the practices with a request that they deal with the information through their usual mechanisms of handling significant events (e.g. discussion at a significant events audit meeting).

If study pharmacists, or member of the study team, have very serious concerns about the performance of a practice in relation to an adverse event, they will discuss this with the Chief Investigator to agree on the most appropriate course of action, which may include a formal report to PCT Clinical Governance Lead. Any such actions will be recorded in project files and the DMEC (Data Monitoring and Ethics Committee for the trial) will be provided with an anonymised report.

Professor Tony Avery

February 2007
