# Supplementary material for: Protocol for the PINCER trial: a cluster randomised trial comparing the effectiveness of a pharmacist-led IT-based intervention with simple feedback in reducing rates of clinically important errors in medicines management in general practices
Source: Trials. 2009 May 1;10:28. doi: 10.1186/1745-6215-10-28 (PMC2685134; doi:10.1186/1745-6215-10-28)
Supplement: Additional file 2 — Pincer Protocol for Publication Appendix 2: Format of tables for publishing the main trial results and within trial economic analysis [file 1745-6215-10-28-S2.doc]

**APPENDIX 2:** **FORMAT OF TABLES FOR PUBLISHING THE MAIN TRIAL RESULTS AND WITHIN TRIAL ECONOMIC ANALYSIS**

The format of tables for publishing the main trial results are shown in the tables below.

**Table 1. Characteristics of practices at baseline by treatment arm.**

| **Practice characteristics** | Intervention arm | Control arm |
| --- | --- | --- |
| Number of practices |  |  |
| Median list size (IQR) |  |  |
| Population by age group (0-14, 15-64, 65-74, ≥ 75) (%) |  |  |
| Median IMD2004 score (IQR) |  |  |
| Training practice (%) |  |  |
| QOF medicines management indicators if available (mean (SD) or median (IQR) number of points per indicator dependent on normality of distribution) |  |  |
| QOF total points (mean (SD) or median (IQR) dependent on normality of distribution) |  |  |

**Table 2. Characteristics of participants at baseline by treatment arm**

| **Participant characteristics** | Intervention arm (%) | Control arm (%) |
| --- | --- | --- |
| Age | Mean (SD) or Median (IQR) | Mean (SD) or Median (IQR) |
| Gender:  Male  Female | n/d  n/d | n/d  n/d |
| Outcome measure 1 | n/d | n/d |
| Outcome measure 2 | n/d | n/d |
| Outcome measure 2 (excluding patients with ischaemic heart disease from the numerator and denominator) | n/d | n/d |
| Outcome measure 3 | n/d | n/d |
| One line for each of the secondary outcome measures reported | n/d | n/d |
| One line for each of the composite outcome measures reported | n/d | n/d |

**Table 3. Prevalence of prescribing and monitoring problems at 6 months follow up by treatment arm.**

| Outcome | Intervention arm (%) | Control arm (%) | Adjusted odds ratio* (95% CI) | ICC |
| --- | --- | --- | --- | --- |
| Primary outcome measures | | | | |
| Outcome measure 1 | n/d | n/d |  |  |
| Outcome measure 2 | n/d | n/d |  |  |
| Outcome measure 2 (excluding patients with coronary heart disease from the numerator and denominator) | n/d | n/d |  |  |
| Outcome measure 3 | n/d | n/d |  |  |
| Secondary outcome measures | | | | |
| One line for each of the secondary outcome measures reported | n/d | n/d |  |  |
| One line for each of the composite outcome measures reported |  |  |  |  |

* Adjusted for randomisation stratum and baseline prevalence of problems

We will present results also adjusted for practice size and deprivation in the text.

**Table 4. Prevalence of prescribing and monitoring problems at 12 months follow up by treatment arm.**

| Outcome | Intervention arm (%) | Control arm (%) | Adjusted odds ratio* (95% CI) | ICC |
| --- | --- | --- | --- | --- |
| Primary outcome measures | | | | |
| Outcome measure 1 | n/d | n/d |  |  |
| Outcome measure 2 | n/d | n/d |  |  |
| Outcome measure 2 (excluding patients with coronary heart disease from the numerator and denominator) | n/d | n/d |  |  |
| Outcome measure 3 | n/d | n/d |  |  |
| Secondary outcome measures | | | | |
| One line for each of the secondary outcome measures reported | n/d | n/d |  |  |
| One line for each of the composite outcome measures reported |  |  |  |  |

* Adjusted for randomisation stratum and baseline prevalence of problems

We will present results also adjusted for practice size and deprivation in the text.

**Table 5. Cost of intervention by treatment arm (within-trial economic analysis)**

| Cost of intervention | Intervention arm mean cost per patient (£) | Control arm mean cost per patient (£) |
| --- | --- | --- |
| Training | n/d | n/d |
| Intervention | n/d | n/d |
| Mean total cost (unadjusted) | n/d | n/d |
| Mean total cost (adjusted) | n/d | n/d |

**Table 6. Summary of disaggregated cost and effectiveness data (within-trial economic analysis)**

|  | Mean errors | | Mean costs (adjusted) | |
| --- | --- | --- | --- | --- |
|  | Intervention arm (%) | Control arm (%) | Intervention arm (£) | Control arm (£) |
| Overall study population |  |  |  |  |
